# As Clinical Markers, Hand-Foot-Skin Reaction and Diarrhea Can Predict Better Outcomes for Hepatocellular Carcinoma Patients Receiving Transarterial Chemoembolization plus Sorafenib

**DOI:** 10.1155/2019/2576349

**Published:** 2019-11-14

**Authors:** Lei Liu, Enxin Wang, Lin Li, Dongyu Chen, Kun Peng, Mengmeng Wang, Guohong Han

**Affiliations:** ^1^Cell Engineering Research Center and Department of Cell Biology, State Key Laboratory of Cancer Biology, Air Force Medical University, Xi'an 710032, China; ^2^Department of Gastroenterology, The Second Affiliated Hospital of Air Force Medical University, Xi'an 710038, China; ^3^Department of Liver Disease and Digestive Interventional Radiology, Xijing Hospital of Digestive Diseases, Air Force Medical University, Xi'an 710032, China; ^4^Department of Medical Imaging, 153th Central Hospital of the Chinese People's Liberation Army, Zhengzhou 450100, China; ^5^Department of Drug and Equipment, Aeromedicine Identification and Training Centre of Air Force, 26 Huaqing Road, Lintong District, Xi'an 710069, China

## Abstract

**Background:**

Combination therapy of transarterial chemoembolization plus sorafenib (TACE-S) has been proven to be safe and effective for hepatocellular carcinoma (HCC); however, this combination therapy is associated with a high incidence of adverse events (AEs). Our study focused on the relationships between AEs and treatment outcomes and aimed to discover AE-based clinical markers that can predict the survival benefits of combination treatment.

**Methods:**

From January 2010 to June 2014, a total of 235 HCC patients treated with TACE-S were retrospectively enrolled. Major sorafenib-related AEs were prospectively recorded, and their correlations with overall survival (OS) were analysed using time-dependent covariate Cox regression analyses.

**Results:**

The majority of the patients (200, 85.1%) were male, and the median age was 51 years old. After two years of follow-up, the median OS of the study population reached 12.4 months. In all, 218 patients (92.8%) presented at least one AE, and 174 (74.0%) suffered AEs ≥2 grade. Based on time-dependent multivariate analyses, the development of hand-foot skin reaction (HFSR) ≥2 grade (HR = 0.43, 95% CI: 0.32–0.58, *P* < 0.001) and diarrhoea ≥1 grade (HR = 0.72, 95% CI: 0.53–0.97, *P*=0.029) were identified as independent predictors of prolonged OS. Moreover, patients who developed both HFSR ≥2 grade and diarrhoea ≥1 grade achieved better outcomes than those patients who developed either or neither of these AEs (HR = 1.51, 95% CI: 1.11–2.06, *P*=0.009).

**Conclusions:**

The development of HFSR ≥2 grade or diarrhoea ≥1 grade during TACE-S treatment indicated prolonged OS, and these AEs should be considered important clinical markers for predicting patient prognoses.

## 1. Background

Hepatocellular carcinoma (HCC), the third leading cause of cancer and the fifth most common malignant tumour, results in 700,000 patient deaths worldwide every year [1]. Surgical resection, liver transplantation, radiofrequency ablation, transarterial chemoembolization (TACE) and sorafenib are major therapies for treating HCC across different Barcelona Clinic Liver Cancer (BCLC) stages. While TACE is the recommended therapy for BCLC-B HCC, sorafenib is the standard targeted treatment for advanced disease [2]. Combination therapy of TACE plus sorafenib (TACE-S) has been investigated in many studies and is indeed an attractive treatment for decreasing the upregulation of VEGF and PDGF after TACE. Hence, HCC recurrence was theoretically reduced [3]. Although the safety of TACE-S has already been proven, its superiority over TACE alone remains controversial.

Several targeted drugs used for cancers, such as erlotinib, cetuximab, and axitinib, can cause AEs such as HFSR, rash, and hypertension. Interestingly, patients who present with those treatment-related AEs achieve better survival than those patients who do not present with AEs [4–12]. Similarly, sorafenib-related AEs that occurred during treatment administration to HCC patients have also been proven to indicate better survival benefits [13–24]. Previous studies focusing on the relationships between AEs and the survival benefits of TACE-S showed that the early appearance of hypertension, HFSR and dermatologic AEs ≥2 grade can serve as clinical markers to predict TACE-S efficacy [25–27].

To some extent, the emergence of AEs reflects the body's response to the treatment. To adjust treatment strategies for patients with a poor response, early surveillance of AEs is of great significance. However, the appearance of AEs is a time-dependent variable. In this study, we aimed to identify clinical markers that can indicate the survival benefits of TACE-S and used time-dependent covariate analyses for more scientific outcomes.

## 2. Methods

### 2.1. Patient Enrolment

From January 2010 to June 2014, 235 HCC patients receiving combination therapy in Xijing Hospital of Digestive Diseases were retrospectively considered. The eligibility criteria were as follows: HCC was diagnosed according to the European Association for the Study of Liver criteria/American Association for the Study of Liver Disease (EASL/AASLD) guidelines, ECOG score of 0 or 1, adequate liver function (Child-Pugh liver function of A to B7; albumin >2.0 g/dl; total bilirubin level <3 mg/dl; ALT and AST <5 times the upper limit of the normal range), adequate cardiac function (controlled hypertension and stable peripheral vascular disease), adequate haematologic function (leukocyte count >3,000 cells/L; platelet count >50 × 10^9^/L; haemoglobin >8.5 g/dl; INR 1-2), and adequate renal function (serum creatinine <1.5 times the upper limit of the normal range; urea nitrogen 3.2–7.1 mmol/L). Patients meeting the following criteria were excluded: Child-Pugh score >7; infiltrative-type HCC; uncontrolled malignant ascites; other accompanying malignant cancer; deficient liver, renal, haematologic or coagulation function; severe cardiac disease such as myocardial infarction over the past year; and fatal damage in other systems. Baseline characteristics of patients, including age, aetiology, and BCLC stage, were collected. All the patients signed the informed consent before enrolment, and the study protocol was approved by the Institutional Review Board of Xijing Hospital.

### 2.2. Treatment

According to the study protocol, treatment decisions were made at the discretion of the institutional multidisciplinary liver tumor board of our center. All participants with unresectable HCC were suitable for sorafenib; and the concomitant TACE was recommended to patients before or after sorafenib administration; finally, the decision was approved by individual patients. Combination therapy was administered with a dose of 400 mg sorafenib twice daily, and the first conventional TACE (c-TACE) procedure was initiated before or after two weeks of sorafenib. Before TACE procedure, the hepatic artery angiography was carried out to evaluate the vascular anatomy and tumour vascularity; during TACE, a vascular catheter was inserted selectively into the tumour-feeding artery with an injection containing a mixture of doxorubicin (10–50 mg) and lipiodol (2–20 ml), followed by an embolization using gelatin sponge particles. When residual viable tumours were confirmed or new lesions developed in patients with adequate liver function, repeated TACE was performed. Dose of sorafenib was modified on the development of adverse events mainly according to the individuals' tolerability. Patients were always encouraged to continue sorafenib therapy, unless the toxicities were too serious to endure. When the intolerable toxicity occurred, a gradual sorafenib dose reduction in a stepwise manner was adopted until individuals considered the severity of adverse events acceptable. Temporary discontinuation was allowed in case of persistent intolerability; and sorafenib restarts in these patients was applied as soon as the toxicity was tolerable. Permanent discontinuation was permitted if the unmanageable or life-threatening adverse events occurred.

### 2.3. Follow-Up

Follow-up was repeated monthly. Laboratory tests and standard contrast-enhanced CT scans were conducted every 4 to 6 weeks according to the modified Response Evaluation Criteria in Solid Tumours (mRECIST) criteria, and were operated by individuals blinded to this trial. A twenty percent increase in the greatest diameter of the target lesion compared with former radiologic assessment or the occurrence of a new lesion was classified as progressive disease (PD). Final follow-up lasted until May 2016. We also recorded the time and reason for death when possible. Patients were considered censored when they were lost to follow-up or still alive at the last follow-up. The time of initial TACE, the interval of the TACE procedure, sorafenib administration, dose modification, recovery, reductions and/or interruptions were recorded concretely.

### 2.4. Adverse Events

AEs that occurred during the treatment process were retrospectively recorded. The occurrence time, severity, and progression or reduction was included. The main related AEs we observed included HFSR, alopecia, rash, diarrhoea, fatigue, voice change, dental ulcers and hypertension, which were recorded and graded by three independent doctors according to the Criteria for Adverse Events version 3.0 (CTCAE v3.0). When disagreements occurred, consensus was achieved through discussion. Stable AEs were recorded every 4 weeks after treatment initiation. Severe AEs were monitored and carefully managed in a timely manner.

### 2.5. Statistical Analysis

Continuous variables were described in terms of the median with interquartile range (IQR). Categorical variables were described in terms of percentages and frequencies. OS was defined as the time from first TACE until the date of death or the last follow-up. Univariate analyses were used to analyse the relationship between baseline characteristics and OS. Cox proportional hazards models were generated to examine risk factors that may have relationships with survival. For the development of AEs, time-dependent Cox regression analyses were applied to rule out the potential time-dependent bias. OS was compared using Kaplan–Meier curves with the log-rank test. Hazard ratios (HRs) and 95% confidence intervals (CIs) were calculated. For all outcomes, a *P* value <0.05 indicated statistical significance. All analyses were conducted by SPSS version 22.0.

## 3. Results

### 3.1. Patient Characteristics

The baseline characteristics of patients are shown in Table 1. Of the 235 included patients, 85% were male, 85.50% were patients infected with HBV, and 96.20% were in Child-Pugh A. Seventy (29.80%) patients were diagnosed at BCLC-B, and 152 (64.70%) were at BCLC-C. In total, two hundred fifteen (91.5%) patients were newly diagnosed with HCC and had not undergone any treatments before. Totally, there were 62 (26.4%) patients with extrahepatic spread (EHS) of HCC and 69 (29.4%) patients with portal vein tumour thrombosis (PVTT). Sorafenib was administered with a median duration of 12.5 (IQR 7.8–22.7) months. The median cycle of TACE was 3 (IQR 1–4). The median OS was 12.4 months (95% CI: 10.4–14.3). Regarding modification of sorafenib dose, forty-one (17.4%) patients had dose reduction, which was mainly because of AEs. Twenty-four patients recovered from 800 mg sorafenib daily after a while. In addition, one hundred ninety-three (82.1%) patients had dose interruption. In the 235 included patients, there were 45 (29.1%) with complete response, 62 (26.4%) with partial response, 95 (40.4%) with stable disease and 33 (14.0%) with progression disease, according to the mRECIST criteria.

### 3.2. Adverse Events

Detailed incidence of sorafenib-related AEs is shown in Table 2. HFSR, alopecia, rash, diarrhoea and fatigue were the five most common AEs. Almost all patients (218, 92.8%) presented at least 1 grade AE during treatment. The majority of patients (174, 74.0%) presented with ≥2 grade AEs, mainly including HFSR, rash, and diarrhoea. The minority of patients (59, 25.1%) presented with AEs ≥3 grade, with the majority presenting with HFSR (19.10%). No grade 4 AEs occurred in any patients. Thirty-six patients (15.32%) needed dose modification due to AEs. Twenty-seven patients (11.49%) needed dose reduction. Thirty-two patients (13.62%) experienced drug interruption. Additionally, the AEs due to TACE mainly consisted of abdominal pain (131, 55.7%), fever (90, 38.3%), nausea (62, 26.4%) and hepatic failure (23, 9.8%).

### 3.3. Clinical Marker Assessment

**Figure 1:**
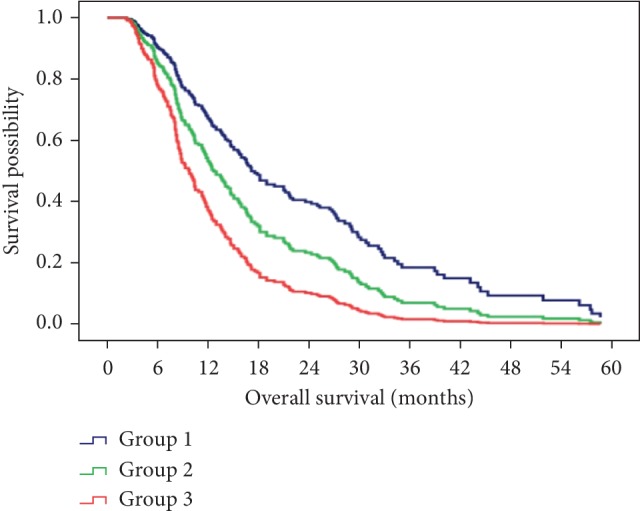
The difference in survival with combination therapy of TACE and sorafenib after dividing patients into 3 groups based on diarrhea and HFSR-response. Group 1, complete responders; Group 2, partial responders; Group 3, non-responders.

According to univariate analyses of baseline variables, the ECOG score, the tumour size, the number of HCC nodules, PVTT, EHS, ascites, the AFP level, the albumin level, the total bilirubin level and the AST level were significantly associated with OS (Table 3). Based on time-dependent univariate analyses of AEs, we found that any AEs ≥1 grade, AEs ≥2 grade, fatigue ≥1 grade or HFSR ≥2 grade showed a significant relationship with OS. After adjustment with significant baseline characteristics using time-dependent multivariate Cox regression analyses, HFSR ≥1 grade (HR = 0.70, 95% CI: 0.50–0.98, *P*=0.038), HFSR ≥2 grade (HR = 0.43, 95% CI: 0.32–0.58, *P* < 0.001) and diarrhoea ≥1 grade (HR = 0.72, 95% CI: 0.53–0.97, *P*=0.029) were statistically significant (Table 4). Considering the HR and *P* value, we finally identified HFSR ≥2 grade or diarrhoea ≥1 grade as independent predictors of the efficacy of TACE-S for HCC patients. Moreover, both HFSR ≥2 grade and HFSR ≥2 grade appeared to indicate better outcomes according to Kaplan–Meier curves (Figure 1). Then, we defined patients who developed both HFSR ≥2 grade and diarrhoea ≥1 grade as complete responders. Patients who experienced either of these AEs were considered partial responders, and patients who experienced neither of these AEs as non-responders. After subgroup analysis, the median OS of the complete responders, partial responders and non-responders were 16.7 months (95% CI: 13.8–19.6), 14.0 months (95% CI: 10.5–17.5), and 7.6 months (95% CI: 5.8–9.5), respectively. The complete responders achieved significantly better survival than those partial responders and non-responders (HR = 1.51, 95% CI: 1.11–2.06, *P*=0.009) (Figure 1).

## 4. Discussion

After the investigation of 235 patients who received TACE-S, HFSR ≥2 grade (HR = 0.43, 95% CI: 0.32–0.58, *P* < 0.001) and diarrhoea ≥1 grade (HR = 0.72, 95% CI: 0.53–0.97, *P*=0.029) were finally identified as independent predictors of prolonged OS based on time-dependent multivariate analysis. Patients with both of these AEs achieved the best survival after combination therapy.

As early as 2004, when Pérez-Soler et al. applied erlotinib treatment for non-small-cell lung cancer (NSCLC), these authors found that the occurrence and severity of rash were associated with survival improvement [4]. Afterwards, several studies observed that other AEs, such as skin toxicity, diarrhoea, and hypertension, can also predict the efficacy of targeted drugs such as cetuximab, axitinib and bevacizumab in treating cancers such as mCRC, HNSCC, and pancreatic cancer [5–7, 9–12]. Sorafenib-related AEs were first found to be survival indicators when treating solid tumours [8]. Many later clinical trials observed that sorafenib-related AEs, including hypertension, HFSR, diarrhoea, alopecia, and fatigue, can indicate the efficacy of sorafenib treatment [13–24]. However, the majority of the mentioned studies used sorafenib treatment alone. A few studies explored whether sorafenib-related AEs can predict the efficacy of combination therapy with sorafenib plus TACE.

In 2016, Zhao et al. found that 2 grade dermatologic AEs within the first month of sorafenib initiation can serve as a clinical marker to predict the efficacy of TACE-S [26]. Zhong et al. found that early onset of hypertension and/or sorafenib-related dermatologic AEs were early biomarkers for the prognosis of patients administered TACE-S [27]. However, AEs are variables that developed after treatment initiation; time-dependent univariate and multivariate analyses should be conducted to rule out lead-time bias. Former studies did not consider this issue. The most original aspect of this study was the conducting of time-dependent multivariate analyses.

In a prospective study conducted by Reig et al. early dermatologic AEs appeared within the first 60 days (DAE60) of treatment, which resulted in dose modification that can predict better survival [23]. A study by Ponziani et al. found that sorafenib dose adjustments can improve the tolerability of relevant AEs, prolong drug exposure and maximize survival [24]. Additionally, the SOFIA study pointed out that patients who received a half dose of sorafenib for more than 70% of the treatment period had better survival than patients who received a full dose for more than 70% of the treatment period or a half dose for less than 70% of the treatment period. A full-dose treatment would be a risk factor for worse patient prognosis [28]. We speculate that moderate or more severe AEs are associated with dose modification and that the more severe the AEs are represented, the stronger reaction the body will have to sorafenib. However, this idea requires the determination of whether survival differs among different AE grades. Yao et al. discovered that long discontinuation of sorafenib treatment might result in disease progression [25].

In future studies, the clinical manifestation and pathogenesis of different AEs should be studied to explore the internal biological mechanism of targeted drug-related AEs. Further, more valuable markers should be explored, and AEs should be quantified according to an authoritative guideline. Then, personalized therapeutic methods where the schedule of drug administration may be individualized will be the tendency. Despite these findings, AEs ≥2 grade can result in an emergency situation; Therefore, moderate AEs should be monitored rigorously. Severe AEs remain a serious issue that needs to be managed properly. Researchers should not pursue further studies of AEs for data collection while ignoring patient safety.

There are several limitations. First, the homogeneity of this retrospective study was not stable, and selection bias regarding patient inclusion might not be completely avoided. Second, 29% of patients were having PVTT and were in BCLC-C stage, who might be harmed by the TACE therapy. Importantly, we included the patients with preserved liver function of Child-Pugh score no more than 7, which ensured those patients to avoid the occurrence of live dysfunction after TACE. In addition, Doyle et al. found that routine sorafenib administration to patients with poor status resulted in a high rate of AEs [29]; this outcome might be the reason why the majority of AEs were of moderate grade. Finally, AE collection was not comprehensive enough; only a few major sorafenib-related AEs were prospectively observed and retrospectively analysed.

In conclusion, the appearance of HFSR ≥2 grade or diarrhoea ≥1 grade was associated with better survival outcomes for HCC patients. These AEs were identified as independent clinical markers for the efficacy of combination therapy.

## Figures and Tables

**Table 1:** Baseline demographics and clinical characteristics of the patients (*N* = 235).

Characteristics	Number (%)/mean ± S.D./median [IQR]
Age (year)	51.3 ± 12.0
Gender (men/women)	200 (85.1)/35 (14.9)
Etiology (HBV/HCV/other)	201 (85.5)/9 (3.8)/25 (10.6)
Child-pugh (A/B)	226 (96.2)/9 (3.8)
ECOG (0/1)	121 (51.5)/114 (48.5)
BCLC (A/B/C)	13 (5.5)/70 (29.8)/152 (64.7)
Previous treatments (yes/no)	20 (8.5)/215 (91.5)
Tumor burden	
Tumor size (cm)	8.5 [6.0–12.3]
No. of HCC nodules	1 [1-2]
PVTT (absent/present)	166 (70.6)/69 (29.4)
EHS (absent/present)	173 (73.6)/62 (26.4)
AFP (ng/mL)	414.5 [12.6–9380.5]
Baseline laboratory values	
Leukocyte (×10E9/L)	5.8 ± 2.7
Hemoglobin (g/L)	137.3 ± 20.5
Platelets (×10E9/L)	144.3 ± 78.6
INR	1.11 ± 0.13
ALT (U/L)	46.0 ± 31.6
AST (U/L)	61.0 ± 43.9
Albumin (g/L)	39.3 ± 5.2
Total bilirubin (*μ*mol/L)	17.5 ± 8.8
Urea nitrogen (mmol/L)	5.0 ± 1.6
Serum creatinine (*μ*mol/L)	83.6 ± 16.6

Abbreviations: S.D., standard deviation; IQR, inter quartile range; HBV, hepatitis B virus; HCV, hepatitis C virus; ECOG, Eastern cooperative oncology group; BCLC, Barcelona clinic liver cancer; PVTT, portal vein tumor thrombus; EHS, extrahepatic spreading; AFP, alpha-fetoprotein; INR, International normalized ratio; ALT, Alanine aminotransferase; AST, Aspartate aminotransferase.

**Table 2:** Number (percentage) of patients reporting nonlaboratory sorafenib related adverse events by CTCAE grading.

Adverse events	Any *n* (%)	Grade 1 *n* (%)	Grade 2 *n* (%)	Grade 3 *n* (%)	Grade 4 *n* (%)
HFSR	182 (77.4)	57 (24.3)	80 (34.0)	45 (19.1)	—
Alopecia	156 (66.4)	139 (59.1)	17 (7.2)	—	—
Rash	124 (52.8)	63 (26.8)	49 (20.9)	12 (5.1)	—
Diarrhea	100 (42.6)	48 (20.4)	41 (17.4)	11 (4.7)	—
Fatigue	102 (43.4)	95 (40.4)	7 (3.0)	—	—
Voice change	46 (19.6)	42 (17.9)	4 (1.7)	—	—
Dental ulcer	26 (11.1)	14 (6.0)	12 (5.1)	—	—
Hypertension	29 (12.3)	15 (6.4)	13 (5.5)	1 (0.4)	—

Abbreviations: CTCAE, common terminology criteria for adverse events; HFSR, hand-foot-skin reaction.

**Table 3:** Univariate analyses of baseline characteristics for overall survival.

Variable	HR (95% CI)	*P* value
Age, per 1 year increase	0.99 (0.98–1.00)	0.197
Gender (ref: female)	0.83 (0.57–1.22)	0.349
Etiology HBV/others (ref: HBV)	0.84 (0.57–1.25)	0.392
Child-pugh A/B (ref: B)	1.53 (0.78–2.99)	0.216
ECOG 0/≥1 (ref: score of 0)	2.54 (1.90–3.39)	<0.001
Previous treatments (ref: no)	0.89 (0.55–1.44)	0.628
Tumor size, per 1 cm increase	1.11 (1.07–1.15)	<0.001
No. of HCC nodules, per 1 lesion increase	1.14 (1.06–1.22)	<0.001
PVTT (ref: absent)	2.98 (2.18–4.07)	<0.001
EHS (ref: absent)	2.02 (1.48–2.75)	<0.001
Ascites (ref: absent)	1.95 (1.22–3.11)	0.005
AFP (ref: ≤400 ng/ml)	1.82 (1.37–2.41)	<0.001
Albumin, per 1 g/L increase	0.96 (0.93–0.99)	0.005
Total bilirubin, per 1 *μ*mol/L increase	1.03 (1.02–1.05)	<0.001
ALT, per 1 U/L increase	1.00 (1.00–1.01)	0.832
AST, per 1 U/L increase	1.01 (1.00–1.01)	<0.001
INR, per 1% increase	1.00 (0.99–1.01)	0.600

Abbreviations: HR, hazard ratio; CI, confidence interval; HBV, hepatitis B virus; ECOG, Eastern Cooperative Oncology Group; HCC, hepatocellular carcinoma; PVTT, portal vein tumor thrombus; EHS, extrahepatic spreading; AFP, alpha-fetoprotein; ALT, Alanine aminotransferase; AST, Aspartate aminotransferase. INR, International normalized ratio.

**Table 4:** Univariate and multivariate analyses of different type of adverse events for overall survival.

Adverse events	Patients (%)	Univatiate analyses		Multivariate analyses	
		Unadjusted HR (95% CI)	*P* Value	Adjusted HR (95% CI)	*P* Value
≥1 grade of adverse events					
Any	218 (92.8)	0.45 (0.26–0.76)	0.003	0.47 (0.27–0.82)	0.008
HFSR	182 (77.5)	0.59 (0.43–0.81)	0.001	0.70 (0.50–0.98)	0.038
Alopecia	156 (66.4)	0.79 (0.59–1.06)	0.122	0.75 (0.55–1.03)	0.076
Rash	124 (52.8)	0.99 (0.75–1.31)	0.944	0.81 (0.61–1.08)	0.154
Diarrhea	100 (42.6)	0.85 (0.64–1.22)	0.478	0.72 (0.53–0.97)	0.029
Fatigue	102 (43.4)	1.34 (1.01–1.77)	0.039	0.95 (0.70–1.28)	0.725
Voice change	46 (19.6)	1.08 (0.76–1.52)	0.679	0.69 (0.46–1.02)	0.062
Dental ulcer	26 (11.1)	0.79 (0.50–1.23)	0.295	0.74 (0.47–1.17)	0.198
Hypertension	29 (12.3)	0.84 (0.56–1.28)	0.425	0.92 (0.60–1.41)	0.704
≥2 grade of adverse events					

Any	174 (74.0)	0.51 (0.38–0.70)	<0.001	0.42 (0.30–0.59)	<0.001
HFSR	125 (53.2)	0.43 (0.33–0.57)	<0.001	0.43 (0.32–0.58)	<0.001
Rash	61 (26.0)	0.89 (0.66–1.22)	0.478	0.75 (0.55–1.04)	0.081
Diarrhea	52 (22.1)	1.02 (0.74–1.41)	0.891	1.07 (0.76–1.50)	0.697
≥3 grade of adverse events					

Any	59 (25.1)	0.73 (0.53–1.01)	0.060	0.65 (0.46–0.91)	0.013
HFSR	45 (19.1)	0.70 (0.48–1.00)	0.051	0.68 (0.47–1.00)	0.051

Abbreviations: HR, hazard ratio; CI, confidence interval; HFSR, hand-foot-skin reaction.

## Data Availability

The author will provide data in time if it requested by the readers.
